# Cook and Move for Your Life, an eHealth intervention for women with breast cancer

**DOI:** 10.1038/s41523-024-00662-2

**Published:** 2024-07-25

**Authors:** Heather Greenlee, Eileen Rillamas-Sun, Rachel L. Yung, Sofia Cobos, Sidney M. Donzella, Yuhan Huang, Liza Schattenkerk, Katherine Ueland, Matthew VanDoren, Samantha A. Myers, Gino Garcia, Theresa King, Margarita Santiago-Torres, Chongzhi Di, Neelendu Dey, Katherine A. Guthrie, Nancy E. Davidson

**Affiliations:** 1 Fred Hutchinson Cancer Center, Seattle, WA USA; 2 University of Washington, Seattle, WA USA

**Keywords:** Breast cancer, Randomized controlled trials

## Abstract

We tested the feasibility and preliminary efficacy of an online diet and physical activity program for women with early-stage breast cancer who had completed surgery, chemotherapy, and radiation therapy (ongoing endocrine therapy allowed). Participants with low fruit and vegetable (F/V) consumption and/or low moderate-to-vigorous physical activity (MVPA) levels were randomized to one of two doses - low (one Zoom group session) or high (12 Zoom group sessions) - of an online lifestyle program with the goal of improving F/V intake and MVPA. All participants received eHealth communications (text messages, study website access), a Fitbit, and a WiFi-enabled scale. Primary objectives evaluated feasibility. Secondary objectives compared the 6-month change in F/V intake and MVPA between the two dose groups. Seventy-four women (mean age = 58.4 years; 87% non-Hispanic White; mean time since diagnosis = 4.6 years) were accrued. Among women in the low dose group, 94% attended the single session; among women in the high dose group, 84% attended at least 8 of the 12 sessions. Retention at 6 months was 93%. High relative to low dose participants consumed 1.5 more servings/day of F/V at 6 months (*P* = 0.007) but MVPA levels did not differ between groups. We successfully implemented an online lifestyle program for early-stage breast cancer survivors. The high dose intervention demonstrated preliminary efficacy in improving F/V consumption in early-stage breast cancer survivors. Future trials can test the intervention in a larger and more diverse population of breast cancer survivors.

## Introduction

Nutrition and physical activity guidelines proposed by the American Cancer Society recommend breast cancer survivors consume five or more servings of fruits and vegetables per day and spend at least 150 min per week in moderate-to-vigorous physical activity (MVPA)^1–3^. Breast cancer recurrence and survival are associated with higher levels of systemic inflammation, greater abdominal adiposity, and reduced physical function, which are all affected by nutrition and physical activity^4,5^. Despite the evidence demonstrating the benefits of these lifestyle behaviors on improving cancer outcomes, most breast cancer survivors do not meet the nutrition and physical activity recommendations^6,7^. Reasons for not meeting these guidelines include a lack of knowledge and/or social support, low self-efficacy, poor self-regulation, and structural barriers for sustainable change^8–11^.

Behavioral health interventions for cancer survivors with high frequency in-person contact are associated with large changes in behaviors, but this mode of delivery is costly and burdensome^12^. Interventions that do not involve face-to-face contact, such as those that are telephone-based or web-based, cost less and can reach more people^12^. Furthermore, evidence indicates technology-based interventions can be a useful and effective strategy for improving health behaviors^13,14^ and are acceptable to cancer survivors^15^. Technologies include eHealth (defined as electronic delivery of health care information) and mHealth, a subcomponent of eHealth involving mobile technologies, such as smartphones and wearable devices. Trials of technology-based health interventions for cancer survivors have not only shown improvements in behavior, but that these interventions are safe and feasible to implement^13,14,16,17^. However, data are limited on the optimal dose, efficacy, and acceptability of multiple delivery modes, and the long-term effectiveness of scalable technology-based interventions^12^.

To help address this gap, we developed and tested the feasibility and preliminary efficacy of the Cook and Move for Your Life study, which offered two scalable doses of a remotely delivered, eHealth behavioral nutrition and physical activity intervention for breast cancer survivors, with the goal of improving diet quality and increasing physical activity. As previously described^18^, the intervention was originally designed as an in-person intervention but due to the COVID-19 pandemic, was changed to a remotely delivered format. If proven effective, this intervention has potential to be cost-effective and to have high population-level reach to support breast cancer survivors in making positive changes to their diet and physical activity behaviors. The study’s primary objective evaluated the feasibility of delivering the online intervention. Secondary objectives compared the effects of two intervention doses on changes in daily intake of fruits and vegetables and minutes per week in MVPA. Exploratory outcomes examined changes in diet quality, weight and body mass index, different physical activity intensities, and levels of physical functioning and anxiety.

## Results

### Sample power and accrual

**Fig. 1:**
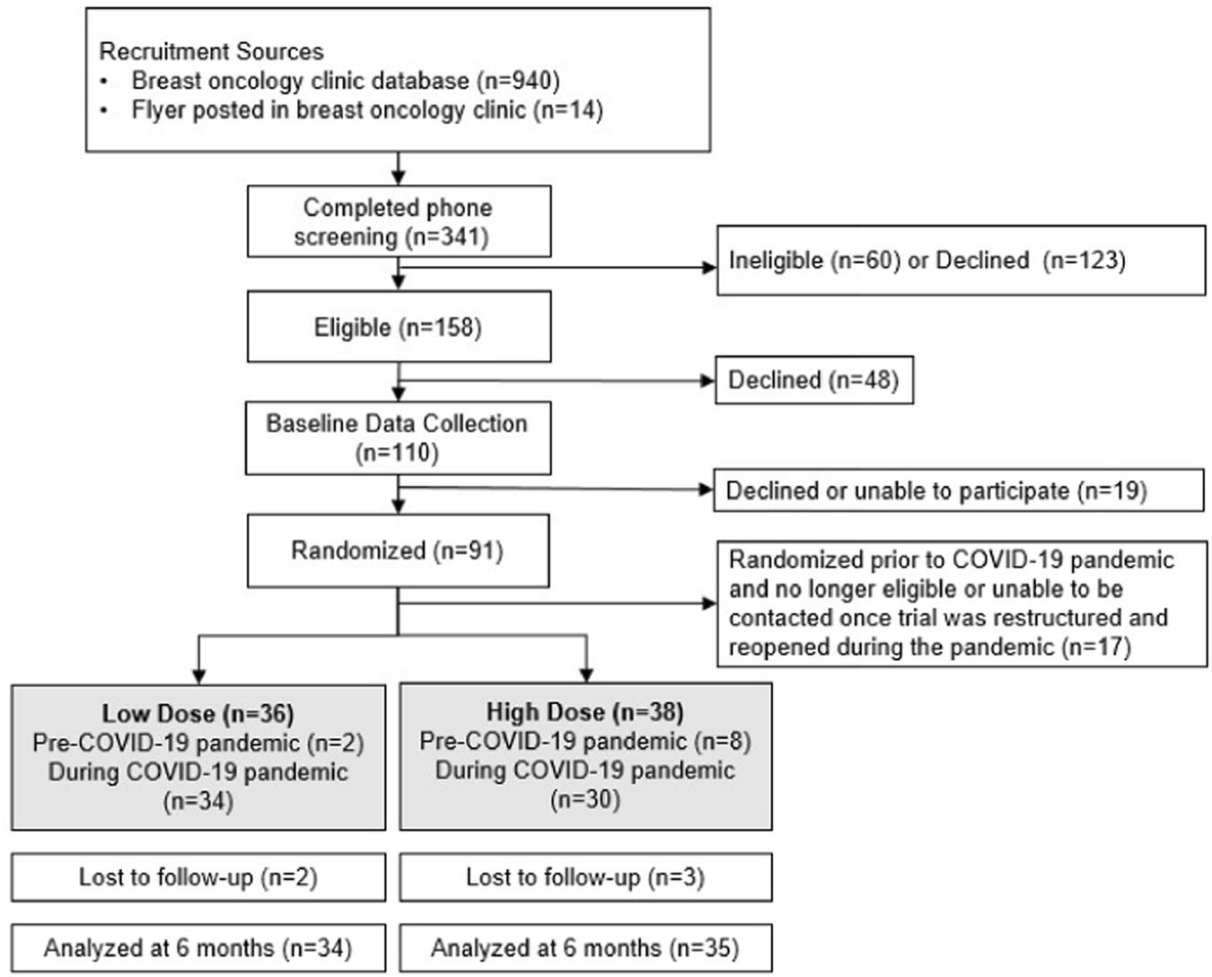
CONSORT diagram for the Cook and Move for Your Life randomized controlled pilot study. Flow chart describing the number of women recruited, ineligible, randomized, and enrolled in the Cook and Move for Your Life study.

From December 2019 to January 2021, a total of 341 breast cancer patients were screened, of which 74 eligible women completed baseline data collection (Fig. 1)^18^. Using permuted block randomization, 38 were randomized to the high dose intervention and 36 were randomized to the low dose intervention. With a minimum adherence of 60% attending the online session, a power calculation indicated that 50 participants (25 per study arm) would provide 81% power to detect a difference of 0.8 standard deviation (SD) units between dose arms. Thus, our enrollment of 36 and 38 women across the study arms provided ample power to test our primary outcomes.

### Baseline characteristics of the study sample

Baseline characteristics are presented in Table 1. Women were a mean (SD) age of 58.4 (10.1) years and had a mean (SD) body mass index of 29.1 (7.3) kg/m^2^. Most women were White (87%), had at least a college degree (65%), were employed (69%), and married (72%). Approximately 90% of women reported being extremely or very comfortable with the internet and text messaging (Table 1). Most (73%) women reported having no major chronic conditions. At baseline, women were an average (SD) of 4.6 (2.3) years since their breast cancer diagnosis and had histories of breast surgery (95%), chemotherapy (50%), radiation therapy (77%), and endocrine therapy (73%). Characteristics at baseline were similar across the two dose interventions.

**Table 1:** Baseline characteristics of Cook and Move for Your Life sample by randomization dose arm

	All (*N* = 74)	High dose arm (*n* = 38)	Low dose arm (*n* = 36)	*p*-value
*Socio-demographic characteristics*				
Age, years, mean (SD)	58.4 (10.1)	59.7 (10.6)	57.0 (9.6)	0.26
Race/Ethnicity, *n* (%)				0.72
White	64 (86.5)	33 (86.8)	31 (86.1)	
Black or African American	1 (1.4)	1 (2.6)	0	
Hispanic	1 (1.4)	1 (2.6)	0	
Asian American/Hawaiian/Pacific Islander	4 (5.4)	2 (5.3)	2 (5.6)	
Mixed Race/Ethnicity	2 (2.7)	0	2 (5.6)	
Prefer not to answer	2 (2.7)	1 (2.6)	1 (2.8)	
Education, *n* (%)				0.29
High school graduate or GED	3 (4.1)	1 (2.6)	2 (5.6)	
Trade school/Associates/Some college	23 (31.1)	15 (39.5)	8 (22.2)	
College degree or higher	48 (64.9)	22 (57.9)	26 (72.2)	
Employment, *n* (%)				0.17
Employed (full-time or part-time)	51 (68.9)	28 (73.7)	23 (63.9)	
Retired	13 (17.6)	6 (15.8)	7 (19.4)	
Homemaker	6 (8.1)	4 (10.5)	2 (5.6)	
Unemployed	4 (5.4)	0	4 (11.1)	
Marital status, *n* (%)				0.45
Married/Living with partner/Common law	53 (71.6)	29 (76.3)	24 (66.7)	
Divorced/Separated	8 (10.8)	5 (13.2)	3 (8.3)	
Widowed	1 (1.4)	0	1 (2.8)	
Single	11 (14.9)	4 (10.5)	7 (19.4)	
Prefer not to answer	1 (1.4)	0	1 (2.8)	
Annual household income, *n* (%)				0.82
$0–$30,000	4 (5.4)	3 (7.9)	1 (2.8)	
$30,001–$60,000	7 (9.5)	3 (7.9)	4 (11.1)	
$60,001–$100,000	17 (23.0)	10 (26.3)	7 (19.4)	
More than $100,000	37 (50.0)	18 (47.4)	19 (52.8)	
Prefer not to answer	9 (12.2)	4 (10.5)	5 (13.9)	
Participates in EBT or SNAP, *n* (%)				0.74
No	72 (97.3)	37 (97.4)	35 (97.2)	
Yes	1 (1.4)	1 (2.6)	0	
Prefer not to answer	1 (1.4)	0	1 (2.8)	
US Born, *n* (%)	68 (91.9)	36 (94.7)	32 (88.9)	0.42
Extremely or very comfortable using the internet, *n* (%)	66 (89.2)	33 (89.2)	33 (97.1)	0.71
Extremely or very comfortable using text messaging, *n* (%)	66 (89.2)	34 (91.9)	32 (94.1)	1
*Clinical characteristics*				
Body mass index, mean (SD)	29.1 (7.3)	29.3 (6.2)	28.8 (8.4)	0.75
Body mass index categories, *n* (%)				0.22
Normal weight, BMI < 25 kg/m^2^	28 (37.8)	12 (31.6)	16 (44.4)	
Overweight, BMI 25 to <30 kg/m^2^	18 (24.3)	8 (21.1)	10 (27.8)	
Obese, BMI ≥ 30 kg/m^2^	28 (37.8)	18 (47.4)	10 (27.8)	
Comorbidity sum (out of 18 possible)				
Mean (SD)	0.4 (0.7)	0.5 (0.8)	0.2 (0.5)	0.07
Median (range)	0 (0–3)	0 (0–3)	0 (0–2)	
Has hypertension, *n* (%)	10 (13.5)	8 (21.1)	2 (5.6)	0.09
Has diabetes, *n* (%)	1 (1.4)	1 (2.6)	0	1
Has cholesterol problems, *n* (%)	9 (12.2)	6 (15.8)	3 (8.3)	0.48
Reports no major medical conditions, *n* (%)	54 (73.0)	25 (65.8)	29 (80.6)	0.15
Years since diagnosis, mean (SD)	4.6 (2.3)	4.7 (2.3)	4.5 (2.2)	0.68
Cancer stage, *n* (%)				0.4
0 (Atypia/In situ)	2 (2.7)	0	2 (5.6)	
I	39 (52.7)	22 (57.9)	17 (47.2)	
II	18 (24.3)	10 (26.3)	8 (22.2)	
III	15 (20.3)	6 (15.8)	9 (25.0)	
Currently on endocrine therapy, *n* (%)	30 (40.5)	17 (44.7)	13 (36.1)	0.45
History of breast surgery, *n* (%)	70 (94.6)	37 (97.4)	33 (91.7)	0.23
History of chemotherapy, *n* (%)	37 (50.0)	19 (50.0)	18 (50.0)	1
History of radiation therapy, *n* (%)	57 (77.0)	28 (73.7)	29 (80.6)	0.34
History of endocrine therapy, *n* (%)	54 (73.0)	28 (73.7)	26 (72.2)	0.94

*EBT* electronic benefits transfer, *GED* general education, *SD* standard deviation, *SNAP* Supplemental Nutrition Assistance Program, *US* United States

### Feasibility by intervention dose arm

The nutrition and physical activity group sessions were well attended by both intervention groups – 84% of high dose participants attended eight or more sessions, while 94% of low dose participants attended their single session (Table 2). Out of 23 possible text message responses requested, women in the low dose intervention were more engaged (mean (SD) number of texts: 18.2 (6.0) vs. 14.9 (6.4); *P* = 0.03). However, women in the high dose arm were more likely to have accessed the study website (84.2% vs. 66.6%, *P* = 0.08). Retention was high across the dose arms, with 92.1% and 97.1% of women in the high and low dose groups (*P* = 0.99), respectively, completing the 6-month questionnaires. Percent of women who returned the accelerometer for 6-month data collection was 84.2% and 72.2%, for high and low dose groups (*P* = 0.21), respectively. Women in the high dose group were more likely than women in the low dose group to report that the nutrition and physical activity group sessions were very or somewhat helpful (90.6% vs. 51.7%, *P* = 0.001). Although women in the low dose arm were more engaged with text messaging, women from both arms reported similar acceptability for helpfulness of texts (Table 2).

**Table 2:** Feasibility measures of Cook and Move for Your Life trial by randomization dose arm

	High dose arm (*n* = 38)	Low dose arm (*n* = 36)	*p*-value
Adherence			
Met attendance study goal^a^, *n* (%)	32 (84.2)	34 (94.4)	0.26
Online sessions attended or viewed, mean (SD)			
Live in real time	7.2 (3.3)	0.9 (0.2)	
Median (Range)	8 (0–11)	1 (0–1)	
Recorded	5.4 (3.5)	0	
Median (Range)	6.5 (0–9)	0	
Either attended live or viewed the recording	9.4 (2.0)	0.9 (0.2)	
Median (Range)	10 (3–12)	1 (0–1)	
Number of text message responses			
Total texts (out of 23 possible), mean (SD)	14.9 (6.4)	18.2 (6.0)	0.025
Goal setting texts (out of 11 possible), mean (SD)	7.1 (3.2)	8.6 (2.9)	0.044
Goal responding texts (out of 11 possible), mean (SD)	7.0 (3.1)	8.8 (2.9)	0.013
Fitbit use			
Wore for ≥1 hour for 70% or more days, *n* (%)	29 (76%)	23 (64%)	0.36
Days wore for ≥1 hour, mean (SD)	139 (52.4)	121 (51.9)	<0.001
Accessed study website, *n* (%)	32 (84.2)	24 (66.6)	0.08
Retention			
Completed 6-month questionnaires, *n* (%)	35 (92.1)	34 (97.1)	0.99
Returned accelerometer for 6-month data collection, *n* (%)	32 (84.2)	26 (72.2)	0.21
Returned biospecimen kit for 6-month data collection, *n* (%)	33 (86.8)	28 (80.0)	0.31
Completed exit interview, *n* (%)	32 (84.2)	29 (80.6)	0.68
Acceptability^b^			
Rated nutrition and physical activity sessions as somewhat or very helpful, *n* (%)	29 (90.6)	15 (51.7)	0.0013
Rated cooking classes as somewhat or very helpful, *n* (%)	28 (87.5)	19 (65.5)	0.07
Rated physical activity training classes as somewhat or very helpful, *n* (%)	28 (87.5)	Question not asked	NA
Rated nutrition text messages somewhat or very helpful, *n* (%)	24 (75.0)	20 (69.0)	0.6
Rated physical activity text messages somewhat or very helpful, *n* (%)	22 (68.8)	21 (72.4)	0.75
Rated eNewsletters as somewhat or very helpful, *n* (%)	13 (40.6)	12 (41.4)	0.95
Rated Fitbit device as somewhat or very helpful, *n* (%)	30 (93.8)	25 (86.2)	0.41

*SD* standard deviation.

^a^At least 8 of 12 sessions for high dose arm or the single session for low dose arm.

^b^Among the *n* = 61 who completed exit interview.

### Diet and physical activity change by intervention dose arm

The mean (SD) fruit and vegetable intake at baseline for women in the high and low dose arms was 5.2 (2.0) and 4.5 (2.7) servings per day, respectively. Over the 6-month study period, relative to women in the low dose arm, women in the high dose arm consumed 1.5 (95% CI: 0.4, 2.6) more servings of fruits and vegetables per day. At baseline, the mean (SD) minutes per week spent in MVPA was 19.5 (18.7) for women in the high dose arm and 25.9 (25.4) for women in the low dose arm. At the 6-month follow-up, women in the high dose arm spent 1.7 (95% CI: −7.0, 10.5) more minutes per day in MVPA compared to women in the low dose arm, although this difference was not statistically significant (Table 3). With regards to other changes in dietary intake observed between arms, women in the high dose relative to the low dose arm, reported consuming 1.1 (95% CI: 0.3, 1.9) more servings of vegetables per day, 170 (95% CI: −321, −18) fewer kilocalories per day, and 11 (95% CI: −20, −2) fewer grams of total fat per day. No statistically significant differences between dose groups were observed for other measures of physical activity, body mass index, weight, physical functioning, or anxiety levels (Table 3).

**Table 3:** Adjusted mean difference in the outcomes associated with being in the high dose intervention arm relative to the low dose intervention arm

	High dose arm, *n* = 38		Low dose arm, *n* = 36			
	Baseline mean (SD)	6-month mean (SD)	Baseline mean (SD)	6-month mean (SD)	Adjusted mean difference (95% CI)^a^	*p*-value
Secondary outcomes						
Fruit and vegetable intake, servings per day	5.2 (2.0)	6.9 (2.6)	4.5 (2.7)	5.2 (2.3)	1.52 (0.44, 2.60)	0.0065
MVPA measured from accelerometer, minutes per day	19.5 (18.7)	24.1 (20.4)	25.9 (25.4)	26.1 (17.7)	1.74 (−6.99, 10.48)	0.69
Exploratory outcomes						
*Dietary intake and quality*						
Fruit intake, servings per day	1.9 (1.4)	2.7 (1.5)	1.2 (1.0)	2.0 (1.4)	0.32 (−0.35, 0.99)	0.34
Vegetable intake, servings per day	3.3 (1.6)	4.2 (1.9)	3.3 (2.3)	3.2 (1.5)	1.11 (0.32, 1.90)	0.006
Energy density, kcal/g	0.57 (0.19)	0.46 (0.15)	0.60 (0.15)	0.55 (0.16)	−0.063 (−0.118, −0.008)	0.026
Total caloric intake, kcal	1706 (445)	1495 (307)	1709 (330)	1669 (421)	−169.5 (−321.3, −17.7)	0.029
Total fat, g	70.9 (23.4)	59.5 (17.7)	73.1 (17.1)	71.8 (24.9)	−10.0 (−0.1, 20.0)	0.048
Total carbohydrates, g	194.8 (59.8)	172.4 (52.5)	192.9 (53.0)	190.1 (60.6)	−17.6 (−38.7, 3.5)	0.1
Total protein, g	70.3 (19.0)	68.4 (14.5)	65.6 (14.8)	63.8 (14.2)	3.0 (−3.4, 9.4)	0.36
Total saturated fatty acids, g	23.1 (10.6)	17.3 (6.3)	25.1 (8.7)	24.2 (11.7)	−4.6 (0.03, −9.3)	0.006
Total monounsaturated fatty acids, g	25.9 (8.4)	23.0 (7.3)	26.5 (6.0)	26.1 (9.5)	−2.9 (−6.8, 1.0)	0.14
Total polyunsaturated fatty acids, g	15.8 (6.6)	14.1 (6.2)	15.8 (6.5)	15.9 (6.1)	1.8 (−4.5, 0.9)	0.18
Total dietary fiber, g	22.9 (7.9)	25.0 (8.8)	19.2 (6.4)	21.6 (7.1)	0.8 (−2.0, 3.5)	0.58
Total sugars, g	73.4 (28.1)	69.0 (25.5)	79.5 (33.7)	75.6 (34.7)	−2.4 (−13.8, 9.1)	0.68
Healthy Eating Index-2015 total score	66.4 (13.6)	75.4 (11.4)	59.6 (11.6)	68.4 (13.8)	3.6 (−1.7, 8.8)	0.18
*Anthropometrics*						
Body mass index, kg/m^2^	29.3 (6.2)	28.5 (5.9)	28.8 (8.4)	28.1 (8.5)	−0.24 (−0.97 0.48)	0.66
Weight, kg	79.0 (15.4)	76.9 (15.2)	78.6 (23.8)	76.8 (24.5)	−0.51 (−2.35, 1.33)	0.63
*Self-reported physical activity*						
Sedentary, minutes per day	398 (271)	275 (204)	378 (214)	350 (418)	−95.5 (−262.3, 71.3)	0.88
Light physical activity, minutes per day	28.1 (33.0)	29.1 (32.0)	30.0 (26.4)	41.0 (43.6)	−10.7 (−26.1, 4.7)	0.17
MVPA, minutes per day	40.4 (48.9)	41.4 (34.2)	43.3 (42.7)	56.6 (60.6)	−12.4 (−37.5, 12.8)	0.78
*Physical activity from accelerometer*						
Sedentary, minutes per day	673 (85)	667 (83)	691 (76)	650 (93)	22.2 (−14.7, 59.1)	0.23
Light physical activity, minutes per day	261 (81)	266 (78)	237 (60)	283 (86)	−24.9 (−58.5, 8.8)	0.14
*PROMIS measures*						
Physical functioning score, mean (SD)	29.6 (7.4)	43.7 (1.7)	28.8 (8.3)	44.3 (1.4)	−0.56 (−1.27, 0.15)	0.12
Anxiety score, mean (SD)	51.4 (8.4)	51.0 (7.3)	48.5 (7.9)	47.6 (6.9)	2.53 (−0.49, 5.55)	0.1

*g* grams, *MVPA* moderate-to-vigorous physical activity, *PROMIS* Patient-Reported Outcomes Measurement Information System, *SD* standard deviation.

^a^Adjusted for baseline measures.

## Discussion

This randomized controlled pilot study evaluated the feasibility and preliminary efficacy of two doses of a remotely delivered eHealth intervention for women with a history of breast cancer. Our results indicated good adherence, retention, and acceptability for both the high and low intervention doses. However, women who received the high dose intervention showed more improvements in dietary intake, including increased daily servings of fruits and vegetables, and reductions in total caloric intake, total fat, and saturated fat. The intervention did not effectively improve physical activity levels. Remotely delivered behavioral interventions can be more cost-effective, less burdensome for both study staff and participants, and have the potential for high-population reach compared to in-person interventions. These pilot findings support the need to test the efficacy of a large-scale remotely delivered eHealth nutrition intervention for women with breast cancer.

The use of eHealth interventions to support improvements in dietary intake and physical activity of cancer survivors has become more frequent in recent years^12–14,19–21^. However, knowledge gaps still exist, such as determining the ideal exposure dose for long-term maintenance of behavior change. Since our intervention implemented two different doses, we offer some insight on this gap. For the high dose intervention, a maximum of 12 sessions were offered via two access points – in real-time or through recordings. Among the 38 women in this arm, eight accessed the sessions solely in real-time and two only viewed the recordings, indicating that most women participated in the sessions using both points of access. Indeed, no one participated in all 12 sessions using only one type of access point, but four did so using a combination. The data also indicated that several women watched some sessions repeatedly, sessions 10 through 12 were not viewed via recording by anyone in the high dose group, and no one from the low dose group viewed the archived recording of the single session. More investigation will be done to understand how these details affected retention, acceptability, and health behavior change. Ultimately, however, these findings suggest that ensuring the intervention is flexible so participants can engage on their own schedule improves adherence. Further, receiving only a single session (per the low dose intervention) was not a sufficient exposure dose – adherence was high, but acceptability of the sessions was low, and no diet or physical activity improvements were observed among women in this dose arm at follow-up.

In addition to the sessions, the eHealth intervention comprised various components delivered over the 6-month intervention period, including weekly motivational text messaging, biweekly electronic newsletters, physical activity self-monitoring using a study-provided Fitbit device, and access to a study website. Study participants from both dose intervention groups had high measures of adherence, study completion, and acceptability of these various study components. These results are consistent with several systematic reviews of eHealth diet and physical activity interventions in cancer survivors^12–14,19–21^. For example, a 2017 systematic review of 11 (six telephone-based, five eHealth-based) remotely delivered behavioral weight loss interventions for female cancer survivors reported 64–75% adherence to self-defined program goals and 70–100% rates of study completion^13^. A more recent (2022) review of 23 diet and physical activity eHealth interventions for cancer survivors reported eight studies using wearable devices with a median wear time of 87%. Additionally, data from four studies using text messaging responses showed a median text response rate of 73%^21^. Moreover, Wang et al. indicated that five studies reported the effectiveness of wearable devices in promoting physical activity engagement and four studies found text messages helped maintain motivation for physical activity^21^. Finally, a systematic review of 27 diet and physical activity eHealth intervention studies for cancer survivors reported that 22 of these studies had good retention, defined as at least 80% of assessments completed for interventions no more than 6-months in duration^12^.

Examination of secondary and exploratory dietary outcomes by intervention dose revealed improvements in dietary intake only among women who received the high dose intervention. However, we observed no change in physical activity or weight measures in either intervention dose. The existing literature indicates that the effectiveness of eHealth interventions to change diet and/or physical activity in cancer survivors is mixed, with some studies improving both behaviors, some affecting only one behavior, and some reporting no statistically significant changes in either diet or physical activity^12,13,21^. There is some consensus that eHealth interventions focusing on one specific behavior, such as fruit and vegetable intake, spending more minutes in MVPA, or weight loss may be more effective than interventions designed to target more than one behavior change simultaneously. Self-reported time spent in MVPA was about twice that of MVPA levels identified from accelerometer and prior studies have shown people overestimate their physical activity when it is self-reported compared to when it is measured objectively^22^, including in cancer survivors^23,24^. This overestimation might have affected the participant’s motivation and need to be more physically active. Of note, an inclusion criterion for study participation was self-reported daily consumption of 5 servings of fruits and vegetables or less or spending less than 150 min per week in MVPA. Despite this, the average consumption of fruit and vegetable intake at baseline for women in the high and low dose group was 5.2 and 4.5 daily servings, respectively. Regarding physical activity levels at baseline, the average minutes per week in MVPA at baseline was 137 and 181 for those in the high and low dose arms, respectively. Thus, women in our study were relatively close to meeting the recommended nutrition and physical activity guidelines proposed by the American Cancer Society at study enrollment. Although women in the high dose intervention significantly improved their fruit and vegetable intake by 1.5 servings per day relative to women in the low dose intervention, by the end of the study, women from both dose arms were, on average, meeting the nationally recommended guidelines for fruit and vegetable consumption and minutes spent in MVPA.

This trial has multiple strengths. The intervention was based on both Social Cognitive Theory and Self-Determination Theory. Lifestyle behavioral interventions based upon theoretical frameworks have been shown to have stronger effects than those that are not theory-based. It is novel to have tested two dose levels of an intervention. The low dose intervention was designed to be like clinical usual care that may offer a one-time class on lifestyle behaviors for cancer survivors with some low-touch eHealth supports (text messaging and a website). The high dose intervention was designed to provide didactic and experiential education. Another strength is the 6-month duration of the intervention as most trials of this nature are limited to 12 weeks^21^. Lastly, the COVID-19 pandemic forced the implementation of a remotely delivered intervention, which ultimately is a more scalable delivery method.

Despite these strengths, this pilot study has limitations. Most of the women enrolled in the study were White with high measures of socioeconomic status based on average level of education and annual household incomes. This limits the generalizability of our results. Furthermore, whether similar results would be observed in more culturally diverse samples or in women who are non-English speaking or with low socio-economic status remains largely unknown. Wang et al. also recognized a lack of racially and economically diverse study samples across the 23 studies reviewed and recommended future studies test eHealth interventions in larger and more diverse populations^21^. The implementation of multiple eHealth components makes it challenging to distinguish which components were effective at influencing positive behavior changes. However, recent technological advances, such as automation of text messaging and remote downloading of data, allow the inclusion of multiple modes of exposure without increasing costs or burden to study staff or participants^12^. Moreover, the systematic reviews evaluated in this paper suggested that incorporating multiple eHealth components, such as online education programs, text messaging, and study-specific websites, is a common and likely approach for future behavioral eHealth interventions^12,13,21^. Finally, a third of the participants did not provide accelerometer data at follow-up and it is possible these women might have had lower levels of physical activity. This would cause an overestimation of physical activity levels in our findings.

In summary, we found that an online intervention delivered twice monthly over 6 months to early-stage breast cancer survivors successfully improved daily servings of fruits and vegetables and decreased caloric intake and total fat consumption. However, the intervention did not yield significant improvements in physical activity. Given the substantial number of cancer survivors in the US (18+ million), scalable interventions are crucial to promote healthy lifestyle behaviors. In our future trials, we will explore scalable, culturally tailored strategies to enhance diet quality and manage weight effectively in women with breast cancer, with specific focus on survivors from underrepresented populations.

## Methods

### Study design

The Cook and Move for Your Life study design and protocol has been previously described^18^. Briefly, Cook and Move for Your Life was a phase two, randomized controlled pilot trial that aimed to test the feasibility and preliminary efficacy of two scalable doses of a remotely delivered nutrition and physical activity intervention for breast cancer survivors. The low dose arm included one real-time 90-min online group session within the first month of enrollment. The high dose arm offered twelve real-time 90-min online sessions delivered twice monthly over six months. All participants received eHealth communications, including interactive text messages, electronic newsletters, a Fitbit device, a Fitbit Aria WIFI-enabled scale, and access to study-specific password protected and HIPAA compliant webpages on the Fred Hutchinson Cancer Center’s Cook for Your Life website (cookforyourlife.org). Informed consent from all study participants was obtained electronically via REDCap, a HIPAA-compliant platform. The study and protocol complied with all ethical regulations, including the Declaration of Helsinki, and was approved by the Institutional Review Board at the Fred Hutchinson Cancer Center (NCT04200482).

### Study population and recruitment

Women were recruited from Seattle Cancer Care Alliance (now known as Fred Hutchinson Cancer Center) patient database registries or direct physician referral from the breast oncology clinic (Fig. 1). Potential participants were mailed a recruitment letter signed by their physician, then called by phone to gauge interest and screen for eligibility. Women were eligible to participate if they were at least 18 years old, not pregnant, English-speaking, and a non-smoker who had a history of early-stage breast cancer (American Joint Committee on Cancer Stage 0-III with no recurrent or metastatic disease); were at least 60 days post treatment (current endocrine therapy use permitted) and physically functional based on the Eastern Cooperative Oncology Group scores (0-1); had no uncontrolled diabetes or hypertension; self-reported consuming less than five servings of fruits and vegetables per day and/or spending less than 150 min per week in MVPA based on screener questionnaires^25,26^, and had internet access via a smartphone or computer^18^.

### Data collection

Upon receiving their consent, women were mailed the following materials for baseline data collection: 1) an Actigraph GT3X+ accelerometer (Actigraph LLC., Pensacola, FL); 2) a stool and blood collection kit; 3) a Fitbit Aria WIFI-enabled scale (Fitbit Inc., San Francisco, CA); and 4) pre-paid mailers to return the materials back to the study center. To objectively measure physical activity, including time spent in MVPA, participants were asked to wear the accelerometer for seven consecutive days. To evaluate changes in the gut microbiota and systemic inflammation, participants were asked to collect fresh stool and blood samples via the instructions provided. Each Fitbit Aria WIFI-enabled scale was linked to every participant allowing us to objectively capture their weights. In addition, web links were sent to participants for completion of online, REDCap-administered questionnaires. Finally, participants completed three (2 weekday, 1 weekend day) 24-hour dietary recalls administered by study staff over the telephone. These procedures were repeated at the 6-month follow-up, which also included an exit interview.

### eHealth intervention

Upon submitting completed baseline data, a permuted block randomization implemented by our study’s statistician was used to allocate participants to receive the low dose or high dose intervention. The Cook and Move for Your Life intervention was based upon Social Cognitive Theory and Self-Determination Theory theoretical frameworks to target behavioral constructs focused on making positive behavior changes^18^. The low dose intervention included one 90-min nutrition and physical activity group session delivered in the first month of enrollment. The high dose intervention included twelve 90-min nutrition and physical activity education group sessions delivered twice monthly over the 6-month intervention period. The group sessions were from a tailored curriculum focused on improving diet and physical activity knowledge and self-efficacy, addressing barriers to health behavior change, and building skills around cooking, eating, grocery shopping, and engaging in various forms of exercise intensities. Specific topic areas for each session by dose arm have been described^18^. All sessions comprised diet and exercise education, hands-on experiential learning for skills building, and a question-and-answer session. Education and experiential learning components were taught by a registered dietitian, trained chef, culinary educator, and a certified exercise physiologist. All sessions were recorded and made accessible for viewing on the study website according to the participant’s study arm.

To encourage study adherence and retention, all participants, regardless of dose arm assignment, received six months of eHealth communications comprising of weekly motivational texts, biweekly electronic newsletters, a Fitbit activity tracker device for self-monitoring physical activity, and study-specific access to the FHCC-sponsored Cook for Your Life study website. Over the intervention period, 96 text messages were sent to participants, of which 23 requested a response. On alternating weeks, an automated text message was sent asking women to set a nutrition or physical activity goal for that week, which was followed up the next week with another text message asking if the goal was achieved. Alongside eHealth communications and the Fitbit device, all participants also received kitchenware (e.g., chef’s knife, measuring cups, cutting board) and an exercise band (Dyna Band, Bucks, UK) by mail, to promote healthy eating and activity and to enhance study adherence and retention.

At the end of the study, all women randomized to the low dose arm were given online access to all the recorded group sessions from the high dose intervention. Access to the CMFYL study website was provided to all participants for 12 months after the study ended.

### Measures

Feasibility was assessed based on the accrual rate, adherence, retention, and acceptability. The accrual rate was defined as the number of eligible women enrolled. The recruitment goal was 90 women, with an expected enrollment and randomization of 75 women, which accounted for a 17% withdrawal and baseline data incompletion rate based on our previous trials with breast cancer survivors. Adherence was defined as the proportion of women who attended the online group sessions, responded to text messages, wore the Fitbit device, and accessed the study website. High adherence was characterized as 60–70% participation, a threshold based on our experience with similar lifestyle interventions in breast cancer survivors^27,28^. Thus, high adherence to the intervention was attending eight or more of the 12 online sessions among high dose participants and the single session among low dose recipients. Attendance to the sessions was defined as either participating in the real-time group session or viewing the recorded session on the study website, objectively captured via Google Analytics data (Google LLC, Mountain View, CA). Since eHealth communications were the same for both doses, high adherence for text messaging was receiving 14 or more text message responses and, for Fitbit, was wearing the device for at least 70% of the days over the 6-month study period. Retention was defined as the proportion of women with 6-month follow-up data from the online questionnaires, 24-h dietary recalls, accelerometers, and biospecimens, with a prespecified goal of collecting data from 75% or more of the sample. Acceptability was measured from responses to select questions about the intervention asked in the exit interviews.

Dietary intake was calculated using the Nutrition Data System for Research (versions 2016–2019, developed by Nutrition Coordinating Center, University of Minnesota, Minneapolis, MN). Physical activity data was collected by the Actigraph GT3X+ accelerometers, which captured movement across three axes at a frequency of 30 Hz and processed in one-minute epochs using ActiLife V6.13.3 software (Actigraph LLC, Pensacola, FL). Non-wear time was classified using the Choi algorithm for triaxial accelerometers^29^. Valid wear time was defined as having at least 10 h per day of wear for four or more days^29,30^. Only participants who met this wear time criteria were included in the physical activity analysis. MVPA was defined from the ActiLife software.

Demographic, socioeconomic, and clinical characteristics were self-reported at baseline via online questionnaires. Participants also completed measures of self-efficacy and food preference, comfort with technology, physical functioning, and anxiety^31,32^, and self-reported their height and weight. Weight was objectively captured via the WIFI-enabled Fitbit Aria scale. Body mass index was calculated from height and weight. Cancer stage, years since diagnosis, and treatment history were abstracted from participants’ electronic health records.

### Statistical analysis

Participant characteristics and feasibility measures were compared by dose arm using T-tests and Chi-square tests. Normality in the distributions of the outcomes were examined using univariate statistics and histograms; all outcomes were reasonably normal. Using an intent-to-treat approach, differences in 6-month study outcomes between dose arms were estimated from linear regression models as a function of randomization assignment and baseline value of the relevant outcome. A two-sided *p*-value of <0.05 was considered statistically significant and all analysis was programmed using SAS v9.4 (SAS Institute, Cary, NC).

## Data Availability

The datasets used and analyzed for this trial are available from the corresponding author upon reasonable request.

## Code availability

All data were analyzed using SAS v9.4 (SAS Institute, Cary, NC). The SAS programming code used for this trial is available from the corresponding author upon reasonable request.

## References

[1] Clinton, S. K., Giovannucci, E. L. & Hursting, S. D. The World Cancer Research Fund/American Institute for Cancer Research Third Expert Report on Diet, Nutrition, Physical Activity, and Cancer: Impact and Future Directions. *J. Nutr.* **150**, 663–671 (2020). 31758189 10.1093/jn/nxz268 PMC7317613

[2] Rock, C. L. et al. American Cancer Society nutrition and physical activity guideline for cancer survivors. *CA Cancer J. Clin.* **72**, 230–262 (2022). 35294043 10.3322/caac.21719

[3] World Cancer Research Fund/American Institute for Cancer Research. *Diet, Nutrtion, Physical Activity and Cancer: a Global Perspective. Continuous Update Project Expert Report* (2018).

[4] Jochems, S. H. J. et al. Impact of dietary patterns and the main food groups on mortality and recurrence in cancer survivors: a systematic review of current epidemiological literature. *BMJ Open* **8**, e014530 (2018). 29459359 10.1136/bmjopen-2016-014530 PMC5857700

[5] Lahart, I. M., Metsios, G. S., Nevill, A. M. & Carmichael, A. R. Physical activity, risk of death and recurrence in breast cancer survivors: A systematic review and meta-analysis of epidemiological studies. *Acta Oncol.* **54**, 635–654 (2015). 25752971 10.3109/0284186X.2014.998275

[6] Blanchard, C. M., Courneya, K. S. & Stein, K. American Cancer Society’s SCS, II. Cancer survivors’ adherence to lifestyle behavior recommendations and associations with health-related quality of life: results from the American Cancer Society’s SCS-II. *J. Clin. Oncol.* **26**, 2198–2204 (2008). 18445845 10.1200/JCO.2007.14.6217

[7] Harris, H. R., Bergkvist, L. & Wolk, A. Adherence to the World Cancer Research Fund/American Institute for Cancer Research recommendations and breast cancer risk. *Int J. Cancer* **138**, 2657–2664 (2016). 26804371 10.1002/ijc.30015

[8] Coro, D. G., Hutchinson, A. D., Banks, S. & Coates, A. M. Dietary Drivers and Challenges of Australian Breast Cancer Survivors: A Qualitative Study. *Women’s Health Rep.* **3**, 563–572 (2022). 10.1089/whr.2021.0133 PMC9258797 35814608

[9] Ee, C., MacMillan, F., Boyages, J. & McBride, K. Barriers and enablers of weight management after breast cancer: a thematic analysis of free text survey responses using the COM-B model. *BMC Public Health* **22**, 1587 (2022). 35987564 10.1186/s12889-022-13980-6 PMC9392910

[10] Keaver, L. et al. Self-Reported Changes and Perceived Barriers to Healthy Eating and Physical Activity among Global Breast Cancer Survivors: Results from an Exploratory Online Novel Survey. *J. Acad. Nutr. Diet.* **121**, 233–241 e238 (2021). 33109503 10.1016/j.jand.2020.09.031 PMC11217928

[11] Koutoukidis, D. A. et al. Lifestyle advice to cancer survivors: a qualitative study on the perspectives of health professionals. *BMJ Open* **8**, e020313 (2018). 29593021 10.1136/bmjopen-2017-020313 PMC5875617

[12] Goode, A. D., Lawler, S. P., Brakenridge, C. L., Reeves, M. M. & Eakin, E. G. Telephone, print, and Web-based interventions for physical activity, diet, and weight control among cancer survivors: a systematic review. *J. Cancer Surviv* **9**, 660–682 (2015). 25757733 10.1007/s11764-015-0442-2

[13] Harvey, J., Dittus, K. & Mench, E. eHealth and behavioral weight loss interventions for female cancer survivors: A review. *Womens Health* **13**, 80–88 (2017). 10.1177/1745505717731012 PMC7789030 28905688

[14] Roberts, A. L., Fisher, A., Smith, L., Heinrich, M. & Potts, H. W. W. Digital health behaviour change interventions targeting physical activity and diet in cancer survivors: a systematic review and meta-analysis. *J. Cancer Surviv* **11**, 704–719 (2017). 28779220 10.1007/s11764-017-0632-1 PMC5671545

[15] Martin, E. C. et al. Interest in Health Behavior Intervention Delivery Modalities Among Cancer Survivors: A Cross-Sectional Study. *JMIR Cancer* **2**, e1 (2016). 28410164 10.2196/cancer.5247 PMC5369635

[16] Kanera, I. M. et al. Long-term effects of a web-based cancer aftercare intervention on moderate physical activity and vegetable consumption among early cancer survivors: a randomized controlled trial. *Int J. Behav. Nutr. Phys. Act.* **14**, 19 (2017). 28187725 10.1186/s12966-017-0474-2 PMC5303303

[17] Krebs, P. et al. An eHealth Intervention to Increase Physical Activity and Healthy Eating in Older Adult Cancer Survivors: Summative Evaluation Results. *JMIR Cancer* **3**, e4 (2017). 28410171 10.2196/cancer.6435 PMC5392211

[18] Ueland, K. et al. A digital health intervention to improve nutrition and physical activity in breast cancer survivors: Rationale and design of the Cook and Move for Your Life pilot and feasibility randomized controlled trial. *Contemp. Clin. Trials* **123**, 106993 (2022). 36336249 10.1016/j.cct.2022.106993

[19] Dorri, S., Asadi, F., Olfatbakhsh, A. & Kazemi, A. A Systematic Review of Electronic Health (eHealth) interventions to improve physical activity in patients with breast cancer. *Breast Cancer* **27**, 25–46 (2020). 31187411 10.1007/s12282-019-00982-3

[20] Haberlin, C. et al. The use of eHealth to promote physical activity in cancer survivors: a systematic review. *Support Care Cancer* **26**, 3323–3336 (2018). 29909476 10.1007/s00520-018-4305-z

[21] Wang, L. et al. mHealth Interventions to Promote a Healthy Diet and Physical Activity among Cancer Survivors: A Systematic Review of Randomized Controlled Trials. *Cancers* **14**, 3816 (2022). 35954479 10.3390/cancers14153816 PMC9367623

[22] Dyrstad, S. M., Hansen, B. H., Holme, I. M. & Anderssen, S. A. Comparison of self-reported versus accelerometer-measured physical activity. *Med. Sci. Sports Exerc.* **46**, 99–106 (2014). 23793232 10.1249/MSS.0b013e3182a0595f

[23] Biskup, M. et al. Agreement between Accelerometer-Assessed and Self-Reported Physical Activity and Sedentary Behavior in Female Breast Cancer Survivors. *Diagnostics* **13**, 3447 (2023). 37998583 10.3390/diagnostics13223447 PMC10670656

[24] Boyle, T., Lynch, B. M., Courneya, K. S. & Vallance, J. K. Agreement between accelerometer-assessed and self-reported physical activity and sedentary time in colon cancer survivors. *Support Care Cancer* **23**, 1121–1126 (2015). 25301224 10.1007/s00520-014-2453-3

[25] Craig, C. L. et al. International physical activity questionnaire: 12-country reliability and validity. *Med. Sci. Sports Exerc.* **35**, 1381–1395 (2003). 12900694 10.1249/01.MSS.0000078924.61453.FB

[26] Thompson, F. E. et al. Fruit and vegetable assessment: performance of 2 new short instruments and a food frequency questionnaire. *J. Am. Diet. Assoc.* **102**, 1764–1772 (2002). 12487538 10.1016/S0002-8223(02)90379-2

[27] Greenlee, H. et al. inverted exclamation markCocinar Para Su Salud!: Randomized Controlled Trial of a Culturally Based Dietary Intervention among Hispanic Breast Cancer Survivors. *J. Acad. Nutr. Diet.* **115**, 709–723 e703 (2015). 25578926 10.1016/j.jand.2014.11.002 PMC4499508

[28] Greenlee, H. A. et al. A pilot randomized controlled trial of a commercial diet and exercise weight loss program in minority breast cancer survivors. *Obesity* **21**, 65–76 (2013). 23505170 10.1002/oby.20245 PMC4705911

[29] Choi, L., Ward, S. C., Schnelle, J. F. & Buchowski, M. S. Assessment of wear/nonwear time classification algorithms for triaxial accelerometer. *Med Sci. Sports Exerc* **44**, 2009–2016 (2012). 22525772 10.1249/MSS.0b013e318258cb36 PMC3443532

[30] King, W. C., Li, J., Leishear, K., Mitchell, J. E. & Belle, S. H. Determining activity monitor wear time: an influential decision rule. *J. Phys. Act. Health* **8**, 566–580 (2011). 21597130 10.1123/jpah.8.4.566 PMC3711095

[31] Pilkonis, P. A. et al. Item banks for measuring emotional distress from the Patient-Reported Outcomes Measurement Information System (PROMIS(R)): depression, anxiety, and anger. *Assessment* **18**, 263–283 (2011). 21697139 10.1177/1073191111411667 PMC3153635

[32] Rose, M., Bjorner, J. B., Becker, J., Fries, J. F. & Ware, J. E. Evaluation of a preliminary physical function item bank supported the expected advantages of the Patient-Reported Outcomes Measurement Information System (PROMIS). *J. Clin. Epidemiol.* **61**, 17–33 (2008). 18083459 10.1016/j.jclinepi.2006.06.025

